# Mammographic texture features associated with contralateral breast cancer in the WECARE Study

**DOI:** 10.1038/s41523-021-00354-1

**Published:** 2021-11-29

**Authors:** Gordon P. Watt, Julia A. Knight, Christine Lin, Charles F. Lynch, Kathleen E. Malone, Esther M. John, Leslie Bernstein, Jennifer D. Brooks, Anne S. Reiner, Xiaolin Liang, Meghan Woods, Tuong L. Nguyen, John L. Hopper, Malcolm C. Pike, Jonine L. Bernstein

**Affiliations:** 1 Memorial Sloan Kettering Cancer Center, New York, NY USA; 2 Lunenfeld-Tanenbaum Research Institute, Sinai Health, Toronto, ON Canada; 3 Division of Epidemiology, Dalla Lana School of Public Health, Toronto, ON Canada; 4 Penn State College of Medicine, Hershey, PA USA; 5 Department of Epidemiology, University of Iowa, Iowa City, IA USA; 6 Fred Hutchinson Cancer Research Center, Seattle, WA USA; 7 Department of Epidemiology and Population Health, Stanford University School of Medicine, Stanford, CA USA; 8 Department of Medicine, Stanford University School of Medicine, Stanford, CA USA; 9 Beckman Research Institute, City of Hope National Medical Center, Duarte, CA USA; 10 Melbourne School of Population and Global Health, University of Melbourne, Parkville, VIC Australia

**Keywords:** Risk factors, Predictive markers, Breast cancer, Cancer imaging, Cancer epidemiology

## Abstract

To evaluate whether mammographic texture features were associated with second primary contralateral breast cancer (CBC) risk, we created a “texture risk score” using pre-treatment mammograms in a case–control study of 212 women with CBC and 223 controls with unilateral breast cancer. The texture risk score was associated with CBC (odds per adjusted standard deviation = 1.25, 95% CI 1.01–1.56) after adjustment for mammographic percent density and confounders. These results support the potential of texture features for CBC risk assessment of breast cancer survivors.

## Manuscript text

Breast cancer survivors have a high risk of developing a second primary contralateral breast cancer (CBC), between 2 and 6 times greater than the risk of first primary breast cancer in the general population^1^. CBC risk factors include younger age and an estrogen receptor (ER)-negative first breast cancer, family history of breast cancer, and stray radiation dose received by the contralateral breast during treatment for the first breast cancer^1–6^. Mammographic percent density (MPD), among the stronger known predictors for first primary breast cancer, also predicts CBC risk, but the associations are generally weaker than for first primary breast cancer^7,8^. Other quantitative features from mammograms (namely, “texture” features) are possibly associated with first primary breast cancer risk^9,10^, but it is not known whether these features may also identify breast cancer survivors at increased risk of subsequent CBC. In this study, we developed and tested a “texture risk score” for CBC using the Women’s Environment, Cancer, and Radiation Epidemiology (WECARE) Study.

**Fig. 1:**
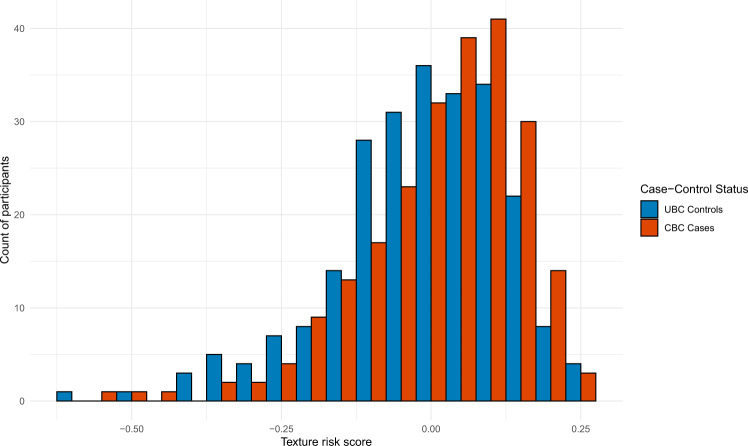
Distribution of texture risk score for unilateral breast cancer (UBC) controls and contralateral breast cancer (CBC) cases in the WECARE Study. WECARE Women’s Environment, Cancer, and Radiation Epidemiology Study, CBC contralateral breast cancer, UBC unilateral breast cancer.

The feature reduction model used to select the most predictive texture features was the LASSO (*λ* = 0.033), which identified three features with non-zero coefficients: gray level run length matrix (GLRLM) short run low gray level emphasis (weight = −0.032); gray level size zone matrix (GLSZM) small area emphasis (weight = −0.093); and GLSZM small area low gray level emphasis (weight = −0.066). The Pearson correlation between the texture risk score and MPD was 0.37 (standard error = 0.04). The score was approximately normally distributed with mean = 0, standard deviation = 0.14, and skewness = −0.98 (see Fig. 1) and was not further transformed. The characteristics of participants with a lower (<median) vs higher (≥median) texture risk score differed by BMI but no other clinical or epidemiological characteristics (Supplementary Table 2).

Table 1 shows that, in multivariable-adjusted models, CBC was statistically significantly associated with the texture risk score for age and BMI (odds ratio per adjusted standard deviation (OPERA) = 1.31, 95% CI 1.06–1.62), and borderline significantly associated with MPD for age and BMI (OPERA 1.21, 95% CI 1.00–1.48). When fitting the texture risk score and MPD together, the association of CBC with the texture risk score remained statistically significant with minimal attenuation while the association with MPD was attenuated by 31% and was no longer statistically significant.

**Table 1:** Association between contralateral breast cancer (CBC), texture risk score, and mammographic percent density (MPD), prior to systemic therapy received during treatment for a first primary breast cancer, in the WECARE II Study (2010–2012).

*N* (cases) = 212 *N* (controls) = 223	OPERA^a^	95% CI	AIC
A. Models including only age- and BMI-adjusted measure			
A1. Texture risk score only	1.27	1.05–1.55	600.8
A2. MPD only	1.14	0.95–1.36	604.9
A3. Texture risk score + MPD	–	–	602.5
Texture risk score	1.24	1.01–1.54	–
MPD	1.06	0.87–1.29	–
B. Models with additional covariate adjustment^b^			
B1. Texture risk score only	1.31	1.06–1.62	604.2
B2. MPD only	1.21	1.00–1.48	606.9
B3. Texture risk score + MPD	–	–	604.7
Texture risk score	1.25	1.01–1.56	–
MPD	1.14	0.93–1.40	–

*WECARE* Women’s Environment, Cancer, and Radiation Epidemiology Study, *BMI* body mass index, *OPERA* odds per age- and BMI-adjusted standard deviation, *CI* confidence interval, *AIC* Akaike’s information criterion.

^a^ Estimated via logistic regression of case–control status on predictors (texture risk score and/or MPD) expressed as odds per age- and BMI- adjusted standard deviation (OPERA).

^b^ Estimated as in (A) with additional adjustment for (A) known risk factors for CBC: age at time of diagnosis of first breast cancer (continuous), estrogen receptor status of first breast cancer (Negative/Positive), stage at diagnosis of first breast cancer (I/II), chemotherapy (Yes/No), hormonal therapy (Yes/No), and radiation therapy (Yes/No) for first breast cancer, menopausal status at time of mammogram (Premenopausal/Postmenopausal), and family history of breast cancer (Yes/No); and (B) matching variables to account for individually matched study design: cancer registry of recruitment, race/ethnicity (Non-Hispanic White vs Other), and year of diagnosis (continuous) of first breast cancer.

From stratified model fits, the association of CBC with the texture risk score was statistically significant for premenopausal women (OPERA 1.39, 95% CI 1.08–1.81), but not for postmenopausal women (OPERA 0.95, 95% CI 0.60–1.48); the OPERA estimates did not differ statistically from one another (*P*-heterogeneity = 0.1). The association of CBC with the texture risk score was greater for women with body mass index (BMI) ≥ 25 kg/m^2^ (1.86, 95% CI 1.23–2.94) compared with women with BMI < 25 kg/m^2^ (1.07, 95% CI 0.82–1.40; *P*-heterogeneity = 0.03) (Table 2).

**Table 2:** Association between CBC risk, texture risk score, and mammographic percent density (MPD), prior to systemic therapy received during treatment for a first primary breast cancer, in the WECARE II Study (2010–2012), stratified by menopausal status and BMI at time of mammogram.

Subgroup	Measure	OPERA^a^	95% CI	Heterogeneity *P*-value^b^
*Menopausal status at time of mammogram*				
Premenopausal (*N* = 319)	Texture risk score	1.39	1.08–1.81	0.1
	MPD	1.19	0.93–1.51	–
Postmenopausal (*N* = 116)	Texture risk score	0.95	0.60–1.48	–
	MPD	1.05	0.71–1.55	–
*BMI (kg/m*^*2*^*) prior to first breast cancer diagnosis*				
<25 (*N* = 265)	Texture risk score	1.07	0.82–1.40	0.03
	MPD	1.07	0.83–1.37	–
≥25 (*N* = 170)	Texture risk score	1.86	1.23–2.94	–
	MPD	1.25	0.88–1.78	–

*CBC* contralateral breast cancer, *WECARE* Women’s Environment, Cancer, and Radiation Epidemiology Study, *BMI* body mass index, *OPERA* odds per age- and BMI-adjusted standard deviation, *CI* confidence interval.

^a^Estimated via logistic regression of case-control status on predictors (texture risk score and mammographic percent density) expressed per as age- and BMI- adjusted standard deviation (OPERA), with additional adjustment (excluding strata variable) for (A) known risk factors for CBC: age at time of diagnosis of first breast cancer (continuous), estrogen receptor status of first breast cancer (Negative/Positive), stage at diagnosis of first breast cancer (I/II), chemotherapy (Yes/No), hormonal therapy (Yes/No), radiation therapy (Yes/No) for the first breast cancer, menopausal status at time of mammogram (Premenopausal/Postmenopausal), and family history of breast cancer (Yes/No); and (B) matching variables to account for matched recruitment: cancer registry of recruitment, race/ethnicity (non-Hispanic White vs other), and year of diagnosis of first breast cancer (continuous).

^b^*P*-value for a likelihood ratio test of the interaction between texture risk score and strata variable.

This study evaluated the association of quantitative mammographic features with CBC risk for breast cancer survivors, whose absolute risk of CBC is high^1^. We found that the combination of three mammographic texture features was associated with CBC after adjusting for known CBC risk factors. Importantly, fitting the texture risk score in a model with MPD strongly attenuated the association between CBC and MPD. This finding is consistent with previous studies of first primary breast cancer, where inclusion of new mammogram-based risk measures, such as texture features, resulted in the associations with conventional MPD attenuating to the null^11,12^. While increased MPD is consistently one of the strongest known risk factors (ORs > 2.0 per SD) for first primary breast cancer, the association with CBC is generally weaker, as reported in the WECARE Study and elsewhere^7,8,13^.

The association of CBC with our texture risk score was essentially unchanged after adjusting for MPD, suggesting that this score, though weakly correlated with MPD, is capturing independent sources of variation in CBC risk that are potentially causal for breast cancer risk^9,14^. Additionally, we identified possible effect modification of the association between CBC and the texture risk score by BMI, which is consistent with BMI-modified associations between mammographic density and first primary breast cancer^15^.

Strengths of this study include control for epidemiological and clinical risk factors of CBC, as well as the use of mammograms taken prior to receiving systemic therapy for the first primary breast cancer, which ensured our findings were not influenced by treatment-related changes to the breast. There were several limitations, however. First, this study lacks external validation of the texture risk score for CBC risk. We attempted to reduce the probability of overfitting by selecting the feature reduction method based on a random subset of the data, as well as by developing only one risk score, and we hope to evaluate this risk score in other large imaging studies of CBC risk when feasible. Second, the WECARE Study recruited participants during the era of film mammograms, which are rarely used today. However, previous studies comparing mammogram-based measures on film and digital images have found little difference in measuring breast size, mammographic density, and risk associations using texture features^16,17^. Third, while the WECARE Study is the largest imaging study of CBC risk with extensive clinical and epidemiological data, we were unable to conduct well-powered subgroup analyses.

In conclusion, mammographic texture features—which are objective and can be generated automatically—are a promising approach to improve risk stratification and tailor surveillance for CBC beginning at the time of the first primary cancer diagnosis.

## Methods

The WECARE Study is a multicenter, population-based, individually matched, case-control study of CBC^18^. Case women were diagnosed with a first primary invasive breast cancer followed ≥1 year later by a second primary invasive cancer in the contralateral breast. Control women were diagnosed with a first primary unilateral invasive breast cancer (UBC) but did not have a second primary invasive CBC and were selected such that each matched control had similar time at risk for CBC (“at-risk time”) as her matched case, with additional matching on age, year of diagnosis, race/ethnicity, and recruitment center. After providing informed consent, participants completed a structured questionnaire and study staff completed medical record abstraction. In the second recruitment phase, the WECARE II Study (2010–2012), participants identified from four population-based cancer registries (Iowa, Seattle, Northern California, and Ontario) also provided consent to access their mammogram of the unaffected breast taken prior to systemic treatment for the first breast cancer. The WECARE Study did not collect the characteristics of facilities where the mammograms were obtained. Nearly all images were film mammograms, which were digitized at two study sites (Seattle and Toronto) using two Kodak Lumisys Digital Scanners set to 12-bit gray-scale resolution with regular calibration (see Supplementary Methods for details). MPD was estimated centrally in Toronto for each film mammogram using the semi-automated thresholding software, Cumulus^19^. All study participants provided written informed consent and the study was approved by the institutional ethics review boards at the University of Iowa (IRB-01), Fred Hutchinson Cancer Research Center, Cancer Prevention Institute of California, Mount Sinai Hospital, and Memorial Sloan Kettering Cancer Center and by the Committee for the Protection of Human Subjects of the State of California.

A fully automated analysis pipeline was used to calculate texture features from digitized cranio-caudal view film mammograms taken prior to systemic therapy for the first breast cancer. Automated in-house software was used to identify the breast area mask (i.e. region for analysis). The range of gray levels within the mask were binned (discretized) into eight gray levels based on intensity quantiles, rather than absolute intensity, to account for possible between-image brightness differences attributable to image acquisition and digitization^9^. From the discretized masks, we calculated five texture matrices: the gray level co-occurrence matrix (GLCM), the gray level run-length matrix (GLRLM), the gray level size zone matrix (GLSZM), the neighborhood gray tone difference matrix (NGTDM), and the neighborhood gray level dependence matrix (NGLDM), with detailed extraction parameters provided in Supplementary Table 1. Seventy-seven unique features, as defined by the Image Biomarker Standardization Initiative^20^, were calculated for the entire breast area from these matrices. Features were centered prior to analysis. Calculation of matrices and features was conducted using the open-source MATLAB package, CERR^21^.

To identify combinations of features associated with CBC, we considered three different penalized regression models: ridge (L2 regularization), elastic net (L1 and/or L2 regularization), and lLASSO (L1 regularization). To identify the tuning parameters for each model without overfitting, we selected 70% of CBC cases and UBC controls at random, and for each model type used 10-fold cross-validation repeated 20 times to select a value of *λ* between 10^−3^ and 10^3^ (and, for elastic net, *α* between 0 and 1). After identifying the best tuning parameters for the ridge, elastic net, and LASSO models, we selected one model from the three that produced the lowest unadjusted root mean square error for CBC prediction in the 30% holdout data. The chosen model was used to develop a “texture risk score” calculated for each WECARE Study participant as a linear combination of the scaled feature values multiplied by the coefficients from the selected penalized regression model.

The association between CBC and the texture risk score was examined by fitting a logistic regression model. To enable direct comparison of the texture risk score and MPD, both measures were parameterized as odds ratio per adjusted standard deviation (OPERA) by dividing the age- and body mass index (BMI)-adjusted residuals of each measure for the controls by the standard deviation of the residuals^22^. We evaluated the association between each OPERA measure and CBC risk, and then further adjusted the models for menopausal status at time of mammogram; stage (localized or regional) and ER status at the time of the first diagnosis; receipt of chemotherapy, hormonal therapy, and radiation therapy for the first breast cancer; first-degree family history of breast cancer; age at menarche; and matching factors. Exploratory stratified models were estimated within subgroups defined by menopausal status at time of mammogram and BMI (<25 kg/m^2^ vs ≥25 kg/m^2^) prior to the first diagnosis.

### Reporting summary

Further information on research design is available in the Nature Research Reporting Summary linked to this article.

## Supplementary information

Supplementary Information

Reporting Summary

## Data Availability

The data that support the findings of this study are available from the corresponding author upon reasonable request.

## Code availability

Access to analytic code repository will be provided upon request.
