# Supplementary material for: Mammographic texture features associated with contralateral breast cancer in the WECARE Study
Source: NPJ Breast Cancer. 2021 Nov 29;7:146. doi: 10.1038/s41523-021-00354-1 (PMC8630158; doi:10.1038/s41523-021-00354-1)
Supplement: Supplementary file 1 — Supplementary Information [file 41523_2021_354_MOESM1_ESM.pdf]

SUPPLEMENTARY INFORMATION

Table of Contents

Supplementary Methods.. 2
Supplementary Table 1... 3
Supplementary Table 2... 4

Supplementary Methods. Description of quality control procedures for digitization of film mammograms in the WECARE II Study.

A. Film mammograms collected at the US-based sites were scanned using equipment at the Fred Hutchinson Cancer Research Center in Seattle. Film mammograms collected in Ontario were scanned using identical equipment and protocols at the University Health Network in Toronto.

B. A Lumisys Digital Scanner (Kodak) was used to create digital images of film mammograms in DICOM and IV2 format (Ontario only) (other file formats might be BMP/Tiff) with a 12-bit gray scale resolution and a pixel pitch of 260 μm (Ontario only; pixel pitch at US sites varied between 174 and 233 μm). During this process, the research personnel ensured that identifying information was visible on the image and that there was a legible label with WECARE Study ID number, view, and laterality.

C. **Calibration scans**. At the start and end of each scanning session at both the Ontario and Seattle sites, a calibration scan was performed as follows. No evidence of drift was observed throughout the study at either location:

    1. Ontario: The calibration film was scanned at the start (pre) and end (post) of each day of digitizing. For each day of digitizing the results of the pre and post calibrations scan were stored to assess systematic differences in optical density (OD).

    2. Seattle: Two calibration protocols were completed. First, a scan of a calibration film was completed at the end of each week of scanning. Second, a randomly selected cranio-caudal view mammogram from that week's batch of mammogram films was re-scanned at the end of each week of scanning. These images were included along with the batch of mammograms uploaded to Ontario for a quality control procedure.
       For the repeat mammogram scans, the mammographic percent density (MPD) of the first scanned image (as measured using Cumulus software) was compared to the MPD (Cumulus) of that same image scanned at the end of the digitizing week.

D. **Image Quality Control** (at each digitization site): After a film was scanned in Toronto and Seattle, the study team checked that no part of the breast was cut off in the digitized image. Other quality control checks included:

    1. Jagged edges along breast edge;
    2. Poor edge visibility along breast edge;
    3. Gridlines;
    4. Label into breast area;
    5. Lines at chest wall, as well as black line at chest wall;
    6. Streaks in image;
    7. Implants;
    8. Presence of guide wires;
    9. Possible copy (not original) mammogram; and
    10. Magnification views.

   If numbers 1 – 6 were observed, the images were re-scanned. If numbers 7 - 11 occurred, a replacement image was identified and scanned with the next batch of films.

E. **Image Quality Control** (in Toronto for images obtained from US sites): Although image quality control was performed by each site during mammogram scanning, additional QC was performed for US-scanned images in Toronto. Each image was assessed using Cumulus as in C2. If any images failed this second QC in Toronto, the respective sites were notified via email and replacement mammograms were located (where possible) and sent for scanning to Seattle and were included in the next batch of mammograms to be scanned. Any replacement images that were found were then re-scanned and underwent the same quality control procedures.

Supplementary Table 1. Parameters used to calculate mammographic texture features in the breast area of digitized mammograms in the WECARE II Study

Feature extraction parameters	Setting	Matrices affected
Number of grey levels	8	GLCM, GLRLM, GLSZM, NGTDM, NGLDM
Directional offsets	0°, 45°, 90°, 135°	GLCM, GLRLM, GLSZM, NGTDM, NGLDM
Averaging method	Average feature value across offsets	GLCM, GLRLM, GLSZM, NGTDM, NGLDM
Pixel offset	1	GLCM
Neighborhood matrix size	3 × 3	NGTDM, NGLDM

Abbreviations. GLCM, gray level co-occurrence matrix; GLRLM, gray level run length matrix; GLSZM, gray level size zone matrix; NGTDM neighborhood gray tone difference matrix; NGLDM, neighborhood gray level distance matrix.

Supplementary Table 2. Characteristics of participants in the WECARE II Study, stratified by texture risk score median

Participant Characteristic	Texture risk score[a] < median, N = 218	Texture risk score ≥ median, N = 217
**Case-control status**		
UBC Controls	124 (57%)	99 (46%)
CBC Cases	94 (43%)	118 (54%)
**Age at time of mammogram, years**		
<45	77 (35%)	88 (41%)
45 to <50	73 (33%)	66 (30%)
≥50	68 (31%)	63 (29%)
**At-risk period[b], years (median, min-max)**	6.5, 2.1-19.3	7.4, 2.1-18.4
**Race/Ethnicity**		
Non-Hispanic White	172 (79%)	183 (84%)
Other	46 (21%)	34 (16%)
**Menopausal status at time of mammogram**		
Premenopausal	158 (72%)	161 (74%)
Postmenopausal	60 (28%)	56 (26%)
**Mammographic Percent Density**		
0-24	94 (43%)	27 (12%)
25-49	107 (49%)	111 (51%)
50-100	17 (8%)	79 (36%)
**ER Status of first breast cancer**		
Positive	150 (69%)	154 (71%)
Negative	68 (31%)	63 (29%)
**Stage at diagnosis of first breast cancer**		
I	145 (67%)	145 (67%)
II	73 (33%)	72 (33%)
**Chemotherapy received for first breast cancer**		
No	69 (32%)	74 (34%)
Yes	149 (68%)	143 (66%)
**Radiation therapy received for first breast cancer**		
No	64 (29%)	77 (35%)
Yes	154 (71%)	140 (65%)
**1st-degree family history of breast cancer**		
No	159 (73%)	156 (72%)
Yes	59 (27%)	61 (28%)
**Body mass index at time of mammogram**		
<25	115 (53%)	150 (69%)
25 to <30	46 (21%)	49 (23%)
30+	57 (26%)	18 (8%)
**Age at menarche, years**		
<13	116 (53%)	115 (53%)
≥13	102 (47%)	102 (47%)

Abbreviations. WECARE, Women's Environment, Cancer, and Radiation Epidemiology Study; UBC, unilateral breast cancer; CBC, contralateral breast cancer; ER, estrogen receptor

[a] Texture risk score based on mammographic texture features selected using LASSO regression in the WECARE II Study

[b] At-risk period is the time between a case's time between their first primary UBC and CBC and an equivalent amount of time for their matched control.
