# Supplementary material for: Mammographic texture features associated with contralateral breast cancer in the WECARE Study
Source: NPJ Breast Cancer. 2021 Nov 29;7:146. doi: 10.1038/s41523-021-00354-1 (PMC8630158; doi:10.1038/s41523-021-00354-1)
Supplement: Supplementary file 2 — Reporting Summary [file 41523_2021_354_MOESM2_ESM.pdf]

nature portfolio

Corresponding author(s): Gordon P. Watt, Ph.D.

Last updated by author(s): 10-18-2021



# Reporting Summary

Nature Portfolio wishes to improve the reproducibility of the work that we publish. This form provides structure for consistency and transparency in reporting. For further information on Nature Portfolio policies, see our Editorial Policies and the Editorial Policy Checklist.

## Statistics

For all statistical analyses, confirm that the following items are present in the figure legend, table legend, main text, or Methods section.

| n/a | Confirmed | |
|---|---|---|
| ☐ | ☒ | The exact sample size (*n*) for each experimental group/condition, given as a discrete number and unit of measurement |
| ☐ | ☒ | A statement on whether measurements were taken from distinct samples or whether the same sample was measured repeatedly |
| ☐ | ☒ | The statistical test(s) used AND whether they are one- or two-sided<br>*Only common tests should be described solely by name; describe more complex techniques in the Methods section.* |
| ☐ | ☒ | A description of all covariates tested |
| ☐ | ☒ | A description of any assumptions or corrections, such as tests of normality and adjustment for multiple comparisons |
| ☐ | ☒ | A full description of the statistical parameters including central tendency (e.g. means) or other basic estimates (e.g. regression coefficient) AND variation (e.g. standard deviation) or associated estimates of uncertainty (e.g. confidence intervals) |
| ☐ | ☒ | For null hypothesis testing, the test statistic (e.g. *F*, *t*, *r*) with confidence intervals, effect sizes, degrees of freedom and *P* value noted<br>*Give P values as exact values whenever suitable.* |
| ☒ | ☐ | For Bayesian analysis, information on the choice of priors and Markov chain Monte Carlo settings |
| ☒ | ☐ | For hierarchical and complex designs, identification of the appropriate level for tests and full reporting of outcomes |
| ☒ | ☐ | Estimates of effect sizes (e.g. Cohen's *d*, Pearson's *r*), indicating how they were calculated |

*Our web collection on statistics for biologists contains articles on many of the points above.*

## Software and code

Policy information about availability of computer code

| | |
|---|---|
| Data collection | Data were collected using standardized paper and online forms using REDCap. Physical copies of film mammograms were collected and centrally digitized (as described in Supplementary Methods) |
| Data analysis | Mammograms were analyzed using Cumulus to estimate mammographic breast density. Texture features were calculated using the open-source MATLAB package CERR. |

For manuscripts utilizing custom algorithms or software that are central to the research but not yet described in published literature, software must be made available to editors and reviewers. We strongly encourage code deposition in a community repository (e.g. GitHub). See the Nature Portfolio guidelines for submitting code & software for further information.

## Data

Policy information about availability of data

All manuscripts must include a data availability statement. This statement should provide the following information, where applicable:

- Accession codes, unique identifiers, or web links for publicly available datasets
- A description of any restrictions on data availability
- For clinical datasets or third party data, please ensure that the statement adheres to our policy

The data that support the findings of this study are available from the corresponding author upon reasonable request.

# Field-specific reporting

Please select the one below that is the best fit for your research. If you are not sure, read the appropriate sections before making your selection.

☒ Life sciences ☐ Behavioural & social sciences ☐ Ecological, evolutionary & environmental sciences

For a reference copy of the document with all sections, see nature.com/documents/nr-reporting-summary-flat.pdf

# Life sciences study design

All studies must disclose on these points even when the disclosure is negative.

| | |
|---|---|
| Sample size | 435 |
| Data exclusions | All participants from the WECARE II Study who provided access to their mammograms were included in the present study. |
| Replication | No third-party dataset exist presently to replicate the findings of an association between texture risk score and CBC. |
| Randomization | Not applicable. |
| Blinding | Cumulus readings of density were blinded to all patient characteristics, including case-control status. The calculation of texture risk score was fully objective requiring no blinding. |

# Reporting for specific materials, systems and methods

We require information from authors about some types of materials, experimental systems and methods used in many studies. Here, indicate whether each material, system or method listed is relevant to your study. If you are not sure if a list item applies to your research, read the appropriate section before selecting a response.

## Materials & experimental systems

| n/a | Involved in the study | |
|---|---|---|
| ☒ | ☐ | Antibodies |
| ☒ | ☐ | Eukaryotic cell lines |
| ☒ | ☐ | Palaeontology and archaeology |
| ☒ | ☐ | Animals and other organisms |
| ☐ | ☒ | Human research participants |
| ☐ | ☒ | Clinical data |
| ☒ | ☐ | Dual use research of concern |

## Methods

| n/a | Involved in the study | |
|---|---|---|
| ☒ | ☐ | ChIP-seq |
| ☒ | ☐ | Flow cytometry |
| ☒ | ☐ | MRI-based neuroimaging |

## Human research participants

Policy information about studies involving human research participants

| | |
|---|---|
| Population characteristics | Women diagnosed with unilateral (local/regional) breast cancer prior to age 55 years of age with at least one year of follow-up. Cases had a second primary breast cancer in the contralateral breast at least one year later; controls were individually matched to cases on time at risk for CBC, age, and recruitment site. |
| Recruitment | Women were identified through the four cancer registries described in the manuscript, and contacted by study staff to invite participation in the study. After explaining the study, eligible participants provided written informed consent to complete an epidemiological questionnaire, provide access to medical records, and provide access to mammograms. Participants must have been living at the time of contact to participate, which may bias the study population toward healthier breast cancer survivors. |
| Ethics oversight | All study participants provided written informed consent and the study was approved by the institutional ethics review boards at the University of Iowa (IRB-01), Fred Hutchinson Cancer Research Center, Cancer Prevention Institute of California, Mount Sinai Hospital, and Memorial Sloan Kettering Cancer Center and by the Committee for the Protection of Human Subjects of the State of California. |

Note that full information on the approval of the study protocol must also be provided in the manuscript.

## Clinical data

Policy information about clinical studies

All manuscripts should comply with the ICMJE guidelines for publication of clinical research and a completed CONSORT checklist must be included with all submissions.

| | |
|---|---|
| Clinical trial registration | N/A |
| Study protocol | N/A |
| Data collection | N/A |
| Outcomes | N/A |
